# Editorial: Genetic mutations in cutaneous malignancies and non-cutaneous diseases

**DOI:** 10.3389/fmed.2022.987494

**Published:** 2022-08-15

**Authors:** Giovanni Paolino, Matteo Riccardo Di Nicola, Carmen Cantisani, Luca Fania, Dario Didona

**Affiliations:** ^1^Unit of Dermatology and Cosmetology, IRCCS Ospedale San Raffaele, Segrate, Italy; ^2^Unità di Dermatologia Clinica, Università Vita-Salute San Raffaele, Milan, Italy; ^3^Unit of Dermatology, Sapienza Medical School of Rome, Policlinico Umberto i Hospital, Rome, Italy; ^4^IDI-IRCCS, Dermatological Research Hospital, Rome, Italy; ^5^Department of Dermatology and Allergology, Philipps University, Marburg, Germany

**Keywords:** skin, genetic, mutations, cutaneous, diagnosis

In the age of personalized medicine, genetic analyses play a pivotal role for the diagnosis, management, and treatment of several cutaneous diseases and malignancies (1). Indeed, considering the skin as an extremely extended organ of the human body, the damage caused by the accumulation of germinal or somatic mutations may be extremely important (2) and several clinicians from different branches should pay close attention to the skin. For this reason, knowing the clinical-pathological and genetic aspects of patients affected by specific mutations can allow the prevention and/or early diagnosis of cutaneous diseases, improving the prognosis and therapeutic setting in these patients. Therefore, we decided to collate a special Research Topic dedicated to this field of research.

Two case reports describe new mutations in some rare cutaneous diseases, improving the diagnosis of these diseases and enlightening their pathogenetic processes. Lofaro et al. describe the case of a female patient with pseudoxanthoma elasticum, who showed cutaneous (papules on neck and axillae as well as marked skin laxity) and ophthalmological (angioid streaks, peau d'orange, retinal pigment epithelium -Bruch's membrane complex abnormalities) manifestations. A previous genetic analysis showed only one ABCC6 pathogenic variant. However, the whole exome sequencing analysis in the same patient showed the presence of rare mutations, such as GGCX and SERPINF1 genes, which contribute to the occurrence of calcification, and ABCA4, AGBL5, CLUAP1, KCNV2 genes, which are mainly involved in ophthalmological alterations. This case highlights how multigene analyses may help clinicians to identify rare sequence variants involved in the diseases, accelerating the diagnostic process, and improving the treatments and relative prognosis. Similarly in a 60-year-old man with Lynch syndrome/Muir-Torre syndrome, Li et al. found a new pathogenic mutation (MSH2 c.1024_1026). The patient had a clear cell carcinoma of the kidney with lung and bone metastases that were successfully treated with chemotherapy. After a disease free survival of about 9 years, the patient developed a rectal neuroendocrine tumor (successfully treated with surgical excision and octreotide acetate microspheres) and multiple cutaneous tumors (benign and malignant tumors, including skin squamous cell carcinoma, keratoacanthoma, sebaceous adenoma, and sebaceous gland carcinoma), which forced the patient to undergo numerous cutaneous surgeries. Furthermore, the patient was treated with sintilimab (anti PD-1) at a dose of 200 mg per 3-week cycle for more than a year without developing new lesions or severe adverse events. Therefore, this case shows that in rare diseases new mutations can be discovered, highlighting important pathogenetic insights to clinicians, and improving in the future the therapeutic options.

The study of genetics in skin diseases can be improved if combined with non-invasive diagnostic methods, such as dermatoscopy and *in vivo* reflectance confocal laser microscopy (RCM), both of which are increasingly used in dermatological clinical practice, as discussed in contributions to this Research Topic. Wang et al. performed a study on dermoscopy and RCM, and gene mutation analysis in a patient with amyloidosis cutis dyschromica (ACD), who showed both cutaneous hypopigmented macules (characterized in dermoscopy by multiple ivory and whitish hypopigmented areas surrounded by brownish blotches) and hyperpigmented macules (characterized in dermoscopy by hyperpigmented spots distributed irregularly). At the same time, RCM showed a highly refractive substance inside the papillary dermis, histologically corresponding to amorphous eosinophilic materials in the papillary dermis. The genetic analysis revealed a non-metastatic melanoma protein B (GPNMB) missense mutations [c.393T>G (p.Y131X; NM_001005340.2)] and a frameshift deletion mutation [c.719_720delTG (p.V240fs; NM_001005340.2)]. This case highlights how the genotype–phenotype correlation can allow the early diagnosis of rare skin diseases. At the same time Paolino et al. performed the analysis of dermatological and dermoscopic baselines in BRCA mutation carriers. The authors found out that in BRCA mutation carriers there is no specific phenotype, in contrast to MCR1R or CDKN2A patients, who typically have red hair or multiple dysplastic nevi, respectively. At the same time, the authors reported that in BRCA mutation carriers there is an increased incidence of eruptive cherry angiomas, without finding any specific association between BRCA mutations and an increased incidence of melanoma. Finally, regarding the dermoscopic analysis of pigmented lesions, the patients showed a prevalence of a reticular pattern, followed by a mixed pattern composed of a central globular or structureless area surrounded by a network. All these results made it possible to identify the cutaneous phenotype of BRCA carriers, highlighting that there is insufficient evidence to increase surveillance in patients with BRCA mutations or with a positive family history for BRCA mutations in the absence of standard cutaneous risk factors.

In their review, Gambini et al. examine basal cell carcinoma (BCC) and hedgehog (Hh) pathway inhibitors. In addition to explaining the pathogenetic mechanisms that lead to the genesis of BCC, the authors highlight the pivotal Hh pathway in BCC proliferation, outlining how a better knowledge made it possible to develop a target therapy with Hh pathway inhibitors, such as vismodegib and sonidegib. This makes the treatment of locally advanced, inoperable, and metastatic BCC possible, and avoids multiple surgical excisions in patients with multiple BCC with or without Gorlin-Goltz syndrome.

The articles in this Research Topic highlight the need for clinical, diagnostic, pathological, and genetic correlations to facilitate the correct diagnosis of patients, meaning personalized treatments can be applied, with better management and a better prognosis. This Research Topic also highlights the importance of multidisciplinary collaboration among different specialists. In conclusion, we thank all 39 authors who contributed to the development of the articles and the reviewers for their valuable contributions. We hope that this Research Topic helps clinicians, researchers, and students in the management of patients with cutaneous diseases carrying specific genetic mutations.

## Author contributions

GP: conceptualization, original draft, review, and editing. MD: review and editing manuscript according to authors guidelines. CC and LF: review and editing. DD: review, editing, and grammar corrections. All authors approved the final version of the manuscript.

## Conflict of interest

The authors declare that the research was conducted in the absence of any commercial or financial relationships that could be construed as a potential conflict of interest.

## Publisher's note

All claims expressed in this article are solely those of the authors and do not necessarily represent those of their affiliated organizations, or those of the publisher, the editors and the reviewers. Any product that may be evaluated in this article, or claim that may be made by its manufacturer, is not guaranteed or endorsed by the publisher.
